# The Isoxazole Derivative of Usnic Acid Induces an ER Stress Response in Breast Cancer Cells That Leads to Paraptosis-like Cell Death

**DOI:** 10.3390/ijms23031802

**Published:** 2022-02-04

**Authors:** Agnieszka Pyrczak-Felczykowska, Tristan A. Reekie, Marcin Jąkalski, Aleksandra Hać, Marcelina Malinowska, Anna Pawlik, Kamil Ryś, Beata Guzow-Krzemińska, Anna Herman-Antosiewicz

**Affiliations:** 1 Department of Physiology, Medical University of Gdańsk, 80-211 Gdańsk, Poland; agnieszka.pyrczak-felczykowska@gumed.edu.pl; 2 School of Chemistry, The University of Sydney, Sydney, NSW 2006, Australia; tristan.reekie@sydney.edu.au; 3 Department of Plant Taxonomy and Nature Conservation, Faculty of Biology, University of Gdańsk, 80-308 Gdańsk, Poland; marcin.jakalski@ug.edu.pl (M.J.); beata.guzow-krzeminska@ug.edu.pl (B.G.-K.); 4 Department of Medical Biology and Genetics, Faculty of Biology, University of Gdańsk, 80-308 Gdańsk, Poland; aleksandra.hac@ug.edu.pl (A.H.); marcelina.malinowska@ug.edu.pl (M.M.); anna.pawlik@ug.edu.pl (A.P.); kamil.rys@phdstud.ug.edu.pl (K.R.)

**Keywords:** usnic acid, anticancer activity, ER stress, IP3R, apoptosis, paraptosis

## Abstract

Derivatives of usnic acid (UA), a secondary metabolite from lichens, were synthesized to improve its anticancer activity and selectivity. Recently we reported the synthesis and activity of an UA isoxazole derivative, named **2b**, against cancer cells of different origins. Herein, the molecular mechanisms underlying its activity and efficacy in vivo were tested. The viability of breast cancer or normal cells has been tested using an MTT assay. Cell and organelle morphology was analyzed using light, electron and fluorescence microscopy. Gene expression was evaluated by RNAseq and protein levels were evaluated by Western blotting. In vivo anticancer activity was evaluated in a mice xenograft model. We found that **2b** induced massive vacuolization which originated from the endoplasmic reticulum (ER). ER stress markers were upregulated both at the mRNA and protein levels. ER stress was caused by the release of Ca^2+^ ions from the ER by IP3R channels which was mediated, at least partly, by phospholipase C (PLC)-synthetized 1,4,5-inositol triphosphate (IP3). ER stress led to cell death with features of apoptosis and paraptosis. When applied to nude mice with xenografted breast cancer cells, **2b** stopped tumour growth. In mice treated with **2b**, vacuolization was observed in tumour cells, but not in other organs. This study shows that the antiproliferative activity of **2b** relates to the induction of ER stress in cancer, not in healthy, cells and it leads to breast cancer cell death in vitro and in vivo.

## 1. Introduction

The rational design of anticancer therapies relies on the identification and the use of the weak points of cancer cells. One of them is endoplasmic reticulum (ER) stress, which is often observed in tumours, and is a result of hypoxia or nutrient depletion, which increase oxidative stress and impair the generation of ATP, thus elevating improperly folded proteins in the ER [1]. Moreover, the constitutive activation of mTORC1 found in many cancers leads to uncontrolled protein synthesis and ER overload [2]. It induces an adaptive response called the unfolded protein response (UPR), which, through activation of ER stress sensors, PERK, IRE1 and ATF6, leads to attenuation of global protein synthesis. Additionally, the translation of a subset of UPR target proteins is elevated, as well as the expression of genes coding for chaperons, foldases and proteins that deal with oxidative stress or are engaged in increased ER volume and secretion [3,4]. Depending on the intensity and duration of ER stress, UPR may promote cell survival or death [5,6,7].

Calcium (Ca^2+^) homeostasis is crucial for the regulation of numerous cellular and physiological processes, including cell growth, proliferation, metabolism and movement [8,9]. Cells maintain their cytosolic concentration of Ca^2+^ at low levels compared with extracellular levels (~100 nM and >1 mM, respectively) thanks to pumps and transporters that extrude Ca^2+^ outside the cell or distribute it for storage into intracellular organelles, such as the ER. The release of Ca^2+^ from the ER is highly regulated and its disturbance may lead to the accumulation of unfolded proteins in the ER lumen and thus induce ER stress [10].

Usnic acid (C_18_H_16_O_7_) (2,6-diacetyl-7,9-dihydroxy-8,9b-dimethyl-dibenzofuran-1,3(2*H*,9b*H*)-dione; UA; *R* isomer = (+)-UA, *S*-isomer = (–)-UA) is a secondary metabolite found in lichens and is especially abundant in the genera *Alectoria*, *Cladonia*, *Evernia*, *Lecanora*, *Ramalina* and *Usnea*. It shows a broad spectrum of biological activities, including antimicrobial, antiviral, antiprotozoal, anti-inflammatory, antiproliferative, anti-metastatic and anti-angiogenic effects, thus garnering interest as an anticancer agent (reviewed in [11,12,13]). UA has been proven to display moderate to strong cytotoxicity against a wide panel of murine and human cancer cells in vitro (reviewed in [14]). For instance, the IC_50_ of (+)-UA in ovarian (A2780), breast (MCF-7 and SKBR-3), colon (HT-29 and HCT116), leukaemia (HL-60 and Jurkat) and cervix (HeLa) cells were in the range of 48.5–199.2 μM (MTT, 72 h) [15]. Studies in vivo showed the promising antitumor activity of UA. For instance, UA (60 mg/kg/day) administered intralesionally for 22 days to mice with xenografted Bcap-37 breast cancer cells stopped tumour growth and angiogenesis [16]. Unfortunately, it appeared to be cytotoxic not only to cancer but also to healthy cells, especially liver cells. The use of UA in food supplements caused cases of intoxication [17,18], and its negative effect on liver cells was observed in vitro and in vivo (reviewed in [19]). For example, treatment of mouse primary hepatocytes with 5 μM UA for 16 h resulted in 98% cell death [20]. Intraperitoneal injections of an UA suspension at a dose of 15 mg/kg/day for 15 days given to male Swiss mice caused hepatic dysfunction, as revealed by an elevated level of serum transaminase and histological observations of necrotic areas in livers [21]. 

The diversity of functional groups within UA makes it an interesting lead compound for the synthesis of derivatives with more favourable biological properties, including higher activity and selectivity toward cancer cells. Early work on structural modification of UA proved that the disruption of the strong intramolecular hydrogen bonds of (–)-UA, which is supposed to increase the water solubility of the compound, failed to generate more active compounds. Moreover, these studies revealed the importance of β-triketone moiety for UA activity [22,23]. Since then, different approaches have been undertaken to improve its bioactivity, including synthetic modifications of UA’s structure [24]. 

Recently, we reported the synthesis of (+)-UA isoxazole derivative **2b**, (*R*)-8-Acetyl-5,7-dihydroxy-3,4a,6-trimethylbenzo[2,3]benzofuro[5,6-d]isoxazol-4(4aH)-one, which is available in a single step from UA [25]. UA derivative **2b** was more toxic than the parent compound to cancer cells of different origins and induced massive vacuolization of MCF-7 breast cancer cells. In this work, we investigated the nature of the vacuoles and a mechanism underlying their appearance in breast cancer cells treated with UA derivative **2b**. Our results indicate that **2b** induces ER stress, which is mediated, at least partly, by the release of Ca^2+^ ions from the ER and leads to cancer cell death, both in vitro and in vivo.

## 2. Results

### 2.1. UA Isoxazole Derivative-Induced Vacuoles in Cancer Cells Are of Endoplasmic Reticulum Origin

**Figure 1:**
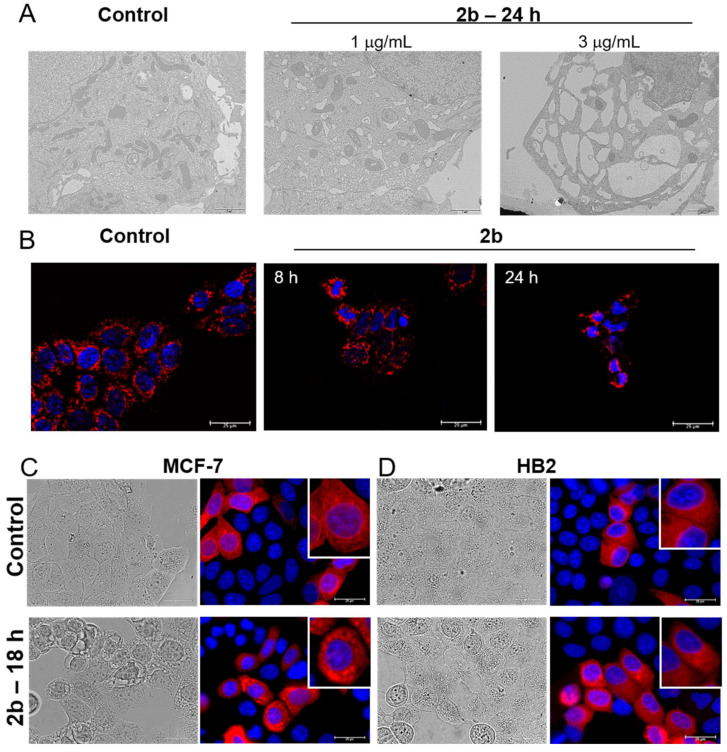
Derivative **2b** induces ER swelling in MCF-7 breast cancer cells but not in noncancerous HB2 breast epithelial cells. (**A**) MCF-7 cells were treated with **2b** (1 or 3 μg/mL) for 24 h and their morphology was examined using transmission electron microscopy. Representative photographs of cells at 1650× magnification are shown. (**B**) Morphology of mitochondria stained with MitoTracker Orange in cells treated or not treated with **2b** (3 μg/mL) for 8 or 24 h and analyzed under a fluorescent microscope (magnification 1000×). MCF-7 (**C**) or HB2 cells (**D**) expressing the ER-retention signal fused with RFP and treated or not treated with **2b** (3 μg/mL) for 18 h and observed under a light or fluorescent microscope (magnification 1000×). Enlarged representative cells are shown in the insets.

Our previous work demonstrated that UA derivative **2b** induced massive cytoplasmic vacuolization (Figure 1A), which was partly reversed by dynasore, an inhibitor of dynamin [25]. To get further insight into the nature of vacuoles, whose size and number increased with the time of exposure to the compound, we visualized mitochondria using MitoTracker Orange and the ER by transfection with CellLight™ ER-RFP, which is a fusion construct of the ER signal sequence of calreticulin, KDEL (ER retention signal) and TagRFP. The morphology of the mitochondria of MCF-7 breast cancer cells was moderately affected by **2b**. They became shorter and rounder than in control cells, especially after 24 h of treatment with 3 μg/mL **2b** (Figure 1B), which is also evident in the TEM pictures (Figure 1A). Interestingly, the **2b** derivative had a pronounced effect on ER morphology. The ER became larger, which was clearly seen after 18 h of exposure to the tested compound. The TagRFP signal colocalized with the large vacuoles seen in the light microscope images (Figure 1C). This indicated that the large vacuoles in **2b**-treated cells are derived from the ER. Importantly, HB2 normal breast cells treated with **2b** at a concentration of 3 μg/mL possessed an ER morphology similar to the control cells (Figure 1D), and their viability was not much affected by **2b** at this concentration (data not shown), which is in line with the lower sensitivity of normal cells to **2b** compared with cancer cells (IC_50_ = 1.3 μg/mL for MCF-7 and about 9 μg/mL for HB2 after 24 h of treatment).

### 2.2. Derivative ***2b*** Induces ER Stress Which Leads to Cell Death

Transcriptome analysis using RNAseq technology was performed to find out the pathways and processes which are affected in MCF-7 cells after 6 or 24 h of treatment with **2b** at the concentration of 3 μg/mL. KEGG analysis showed that protein processing in the ER was the most significantly upregulated pathway by **2b**, both after 6 h and 24 h of treatment (Table 1 and Table 2).

**Figure 2:**
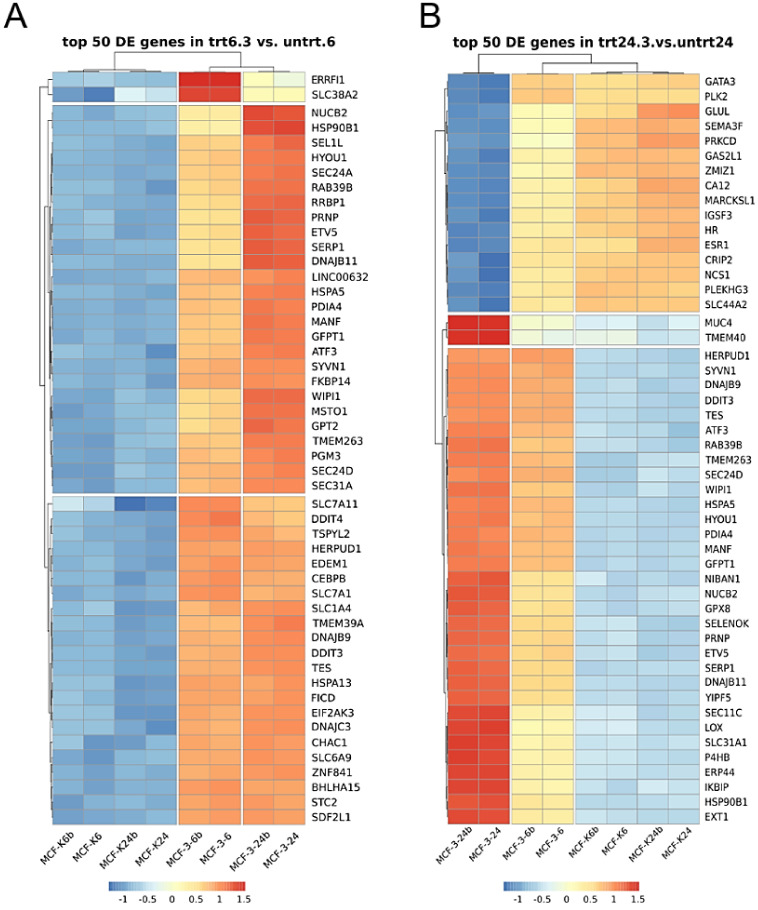
**2b** induces ER stress in breast cancer cells. Heat maps of the top 50 differentially expressed genes in MCF-7 cells treated with 3 μg/mL of **2b** for 6 (**A**) or 24 h (**B**).

**Figure 3:**
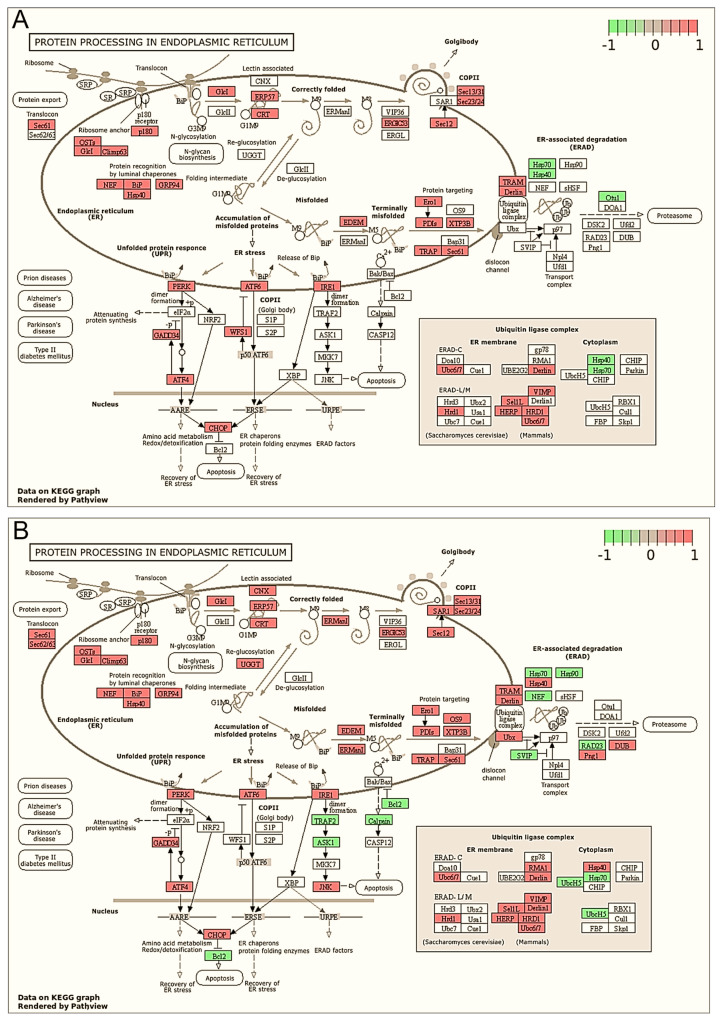
Changes in differentially expressed genes of KEGG pathway related to protein processing in ER after 6-h (**A**) or 24-h (**B**) treatment with **2b** compared with control cells. Downregulated genes appear in green and upregulated genes appear in red.

Upregulated genes related to ER functioning made up 70% and 50% of the top 50 differentially expressed genes in cells exposed to **2b** for 6 or 24 h, respectively, which is shown in heat maps (Figure 2A,B) and in tables (Supplementary data Tables S1 and S2). Among the differentially expressed genes were ER chaperones, such as the glucose-regulated protein GRP78/BIP (a hallmark of UPR), GRP94-endoplasmin and Hsp40; ER stress sensors, such as endoribonuclease, inositol requiring enzyme 1 (IRE1), PKR-like ER kinase (PERK) or ATF6; UPR target genes (ATF4, CHOP, GADD34, ERO1, PDI), components of ERAD (EDEM, Derlin, Ubx and HRD1), and proteins engaged in apoptosis induction (CHOP, JNK, Bcl-2 and DR5) (Figure 3A,B and Supplementary Tables S1 and S2). These results clearly indicate that **2b** induced ER stress. Moreover, gene ontology analyses confirmed that ER stress is one of the most affected biological processes after treatment of MCF-7 cells with **2b**. The results showed that 121 and 155 upregulated genes were annotated to the ER stress response after the 6-h and 24-h treatments, respectively (Supplementary data, Tables S3–S8).

**Figure 4:**
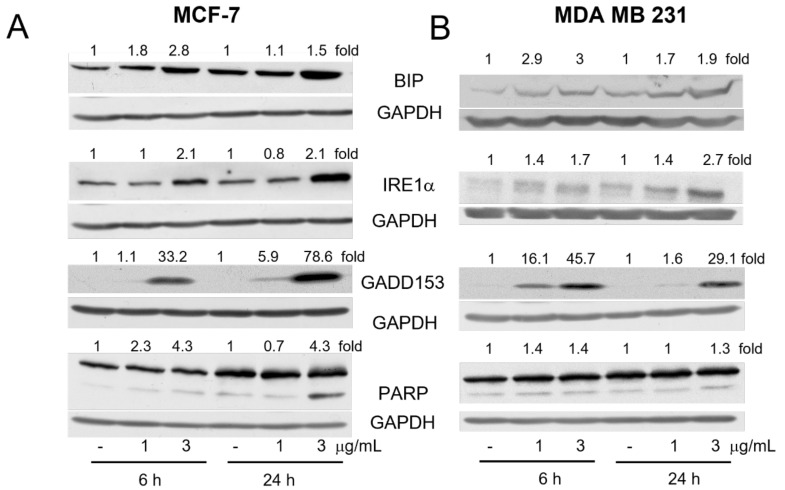
**2b** induces ER stress in breast cancer cells. Immunoblots for ER stress markers, BIP, IRE1α, GADD153 as well as PARP in MCF-7 (**A**) or MDA MB 231 cells (**B**) treated with DMSO or **2b** (1 or 3 μg/mL) for 6 or 24 h. The blots were stripped and reprobed with the anti-GAPDH antibody to ensure equal protein loading. Densitometric scanning data after correction for loading control are above the immunoreactive bands (in the case of PARP quantification of faster migrating, cleaved fragments are shown).

We checked the status of some crucial components of ER stress at the protein level. As shown in Figure 4A, the levels of BIP, IRE1α and GADD153 (CHOP) were increased after 6 h of treatment with **2b**, especially when it was used at the 3 μg/mL concentration. Upregulation of BIP mRNA might indicate the increased activity of the ATF6 transcription factor, and the elevation of GADD153 mRNA indicated the activation of ATF4 and/or ATF6 transcription factors, while the elevation of IRE1α suggested the increased activity of GADD153.

To elucidate whether **2b** induces ER stress in other breast cancer cells, we used the MDA MB 231 cell line representing the triple-negative subtype of breast cancer. As shown in Figure 4B, **2b** upregulated BIP, IRE1α and GADD153 proteins in a dose- and time-dependent manner similarly to that in in oestrogen receptor-positive MCF-7 cells. Caspase-dependent cleavage of PARP was also observed in MDA MB 231 cells, but at a lower level compared with MCF-7 samples, which might be correlated with the lower sensitivity of MDA MB 231 to **2b** (IC_50_ = 3.1 μg/mL after 24 h of treatment).

**Figure 5:**
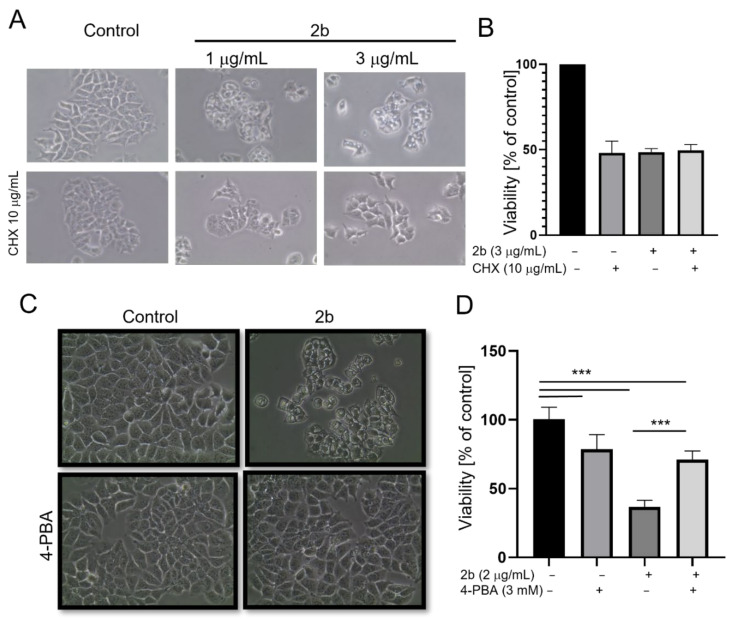
Vacuolization and death of MCF-7 cells are blocked by inhibitors of ER stress. Cells were pretreated with cycloheximide (1 or 10 μg/mL) or 4-PBA (3 mM) for 1 h and then treated with **2b** (1 or 3 μg/mL—(**A**,**B**) or 2 μg/mL—(**C**,**D**) for 24 h. Cell morphology was observed in a light microscope (**A**,**C**) and viability was determined by MTT assay (**B**,**D**). The data are shown as the mean ± SE. Statistical significance was determined by ANOVA followed by Tukey’s multiple comparison test and *** *p* < 0.0001.

As the ER stress is caused by an overload of the ER lumen with improperly folded proteins, which might lead to ER dilatation, we used cycloheximide to abrogate protein synthesis. As shown in Figure 5A, pretreatment with cycloheximide protected against vacuolization and cell morphology changes induced by **2b** after the 24-h exposure. This may also suggest that protein synthesis is necessary for vacuolization, which is one of the features of paraptosis. We did not observe protection against a **2b**-induced drop in viability by cycloheximide; however, it is worth noting that cycloheximide by itself significantly decreased MCF-7 cell viability and did not enhance the cytotoxicity of **2b** (Figure 5B). Importantly, 4-phenylbutyric acid (4-PBA), a small molecular chemical chaperone that prevents protein aggregation and alleviates ER stress-mediated cell damage [26], protected against **2b**-induced vacuolization and drop in cell viability (Figure 5C,D).

### 2.3. ***2b***-Induced ER Stress Is Accompanied by the Release of Calcium Ions

**Figure 6:**
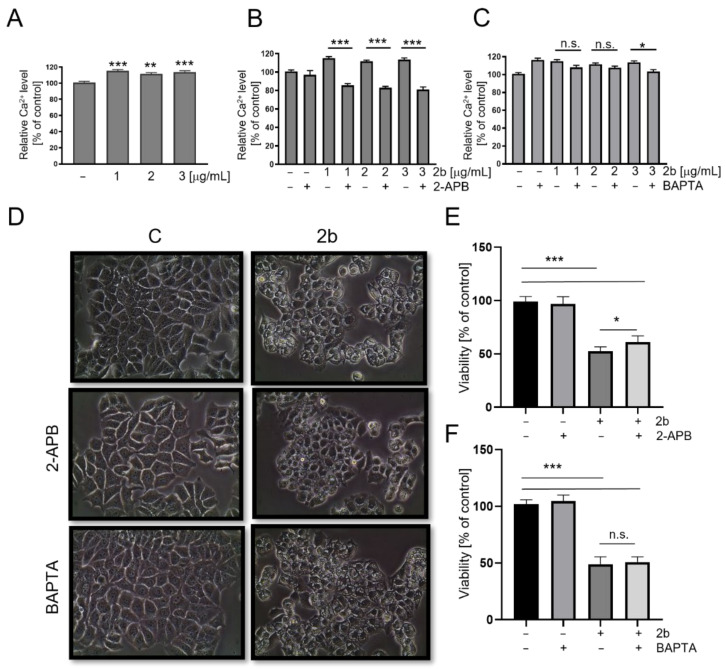
**2b** increased cytosolic Ca^2+^ level through the release of Ca^2+^ from ER. A. MCF-7 cells were treated with **2b** at indicated concentrations and Ca^2+^ level was estimated (**A**). Effect of 2-APB (30 μM) or BAPTA (10 μM) pretreatment on Ca^2+^ level (**B**,**C**, respectively), cell vacuolization (**D**) or viability (**E** and **F**) of cells treated with **2b** was assessed. The data are shown as the mean ± SE. Statistical significance was determined by ANOVA followed by Dunnett’s (**A**) or Tukey’s (**B**–**F**) post hoc tests: * *p* < 0.05, ** *p* < 0.001, *** *p* < 0.0001; n.s.—not significant.

It has been shown that UA induced the elevation of Ca^2+^ in the cytoplasm of hepatic cells, which led to ER stress [27]. To elucidate whether the **2b** derivative acts similarly, we treated MCF-7 cells with **2b** at different concentrations for 6 h, and the calcium level was estimated using the Fluo-4 Direct Assay Kit. As shown in Figure 6A, **2b** increased the amount of cytosolic Ca^2+^ even at the lowest tested concentration. An elevation of cytosolic calcium might be caused by increased uptake from the extracellular environment or depletion of ER stores. To distinguish between these routes, the respective inhibitors were used, i.e., BAPTA (a nonpermeable, selective extracellular calcium chelator) and 2-APB (an inhibitor of IP3 receptors). As shown in Figure 6B, only 2-APB blocked **2b**-induced elevation of cellular calcium. Moreover, it fully protected against vacuolization (Figure 6D) and partly protected against the **2b**-induced drop in cell viability (Figure 6E). Dantrolene, an inhibitor of ryanodine receptors (RyR), another ER calcium release channel, had no effect on the vacuolization and viability drop induced by **2b** (data not shown). These results indicate that it is the deposition of Ca^2+^ from the ER to the cytosol rather than extracellular uptake that is responsible for the elevation of cytosolic Ca^2+^ and the drop in cell survival.

### 2.4. Inhibition of IP3 Synthesis by PLC Protects against Vacuolization and Cell Death Induced by ***2b***

**Figure 7:**
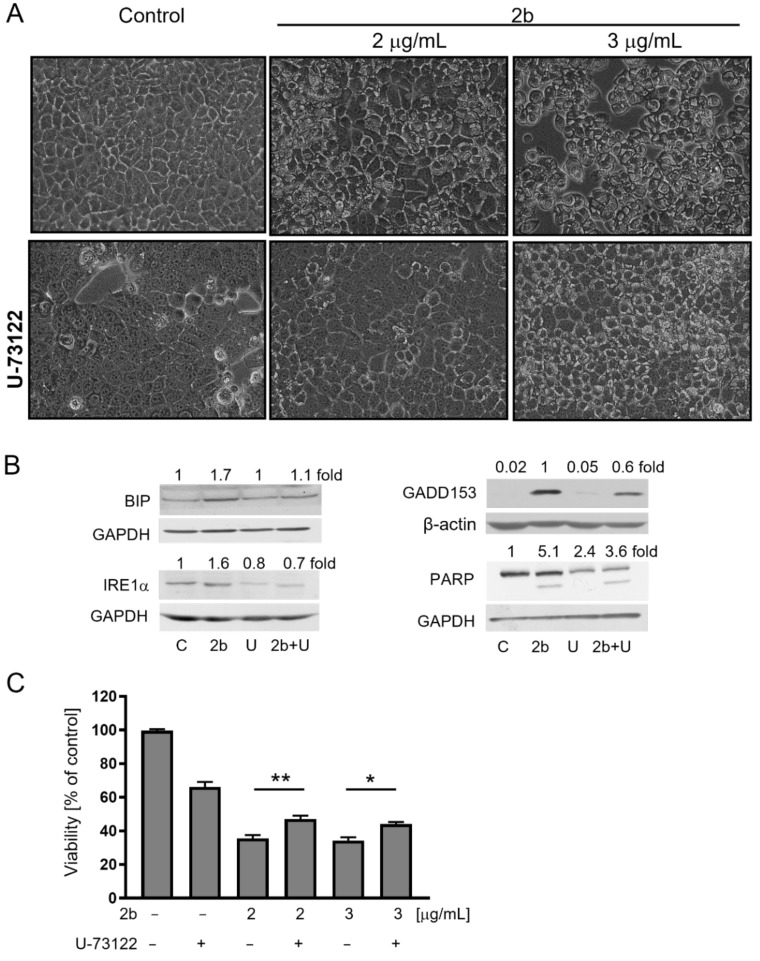
Inhibition of PLC protects against **2b**-induced ER stress and partially—against **2b**-induced cell death. MCF-7 cells were pretreated or not with U73122 (5 μM) for 1 h and then treated with **2b** at indicated concentrations. (**A**) Cell morphology was inspected after 24-h treatment using light microscopy (magnification 20×). (**B**) The levels of ER stress markers were estimated after 6 or 24-h treatment with **2b** (3 μg/mL) by immunoblotting. The blots were stripped and reprobed with an anti-GAPDH antibody to ensure equal protein loading. Densitometric scanning data after correction for loading control are above the immunoreactive bands. (**C**) Viability of cells was evaluated by MTT assay after 24-h treatment with **2b**. The data are shown as the mean ± SE. Statistical significance was determined by one-way ANOVA followed by Tukey’s multiple comparison test: * *p* < 0.05, ** *p* < 0.01.

Activation of IP3 receptors on the ER is regulated by signaling involving phospholipase C (PLC) that generates 1,4,5-inositol triphosphate (IP3) from phosphatidylinositol 4,5-bisphosphate (PIP2) [28,29]. We evaluated the effect of 1-[6-((17b-3-methoxyestra-1,3,5(10)-trien-17-yl)amino)hexyl]-1H-pyrrole-2,5-dione (U-73122), a universal PLC inhibitor, on vacuolization and survival of MCF-7 cells treated with **2b**. Results presented in Figure 7 showed that pre-treatment of cells with U-73122 protected against **2b**-induced vacuolization and an elevation of ER stress markers: BIP, IRE1α, GADD153. It also partially protected against caspase-dependent PARP cleavage and drop in the viability of cells. These results indicate that **2b**, at least partially, acts through PLC-mediated IP3 generation.

### 2.5. Orally Administered ***2b*** Retards MCF-7 Xenografts Growth in Mice

**Figure 8:**
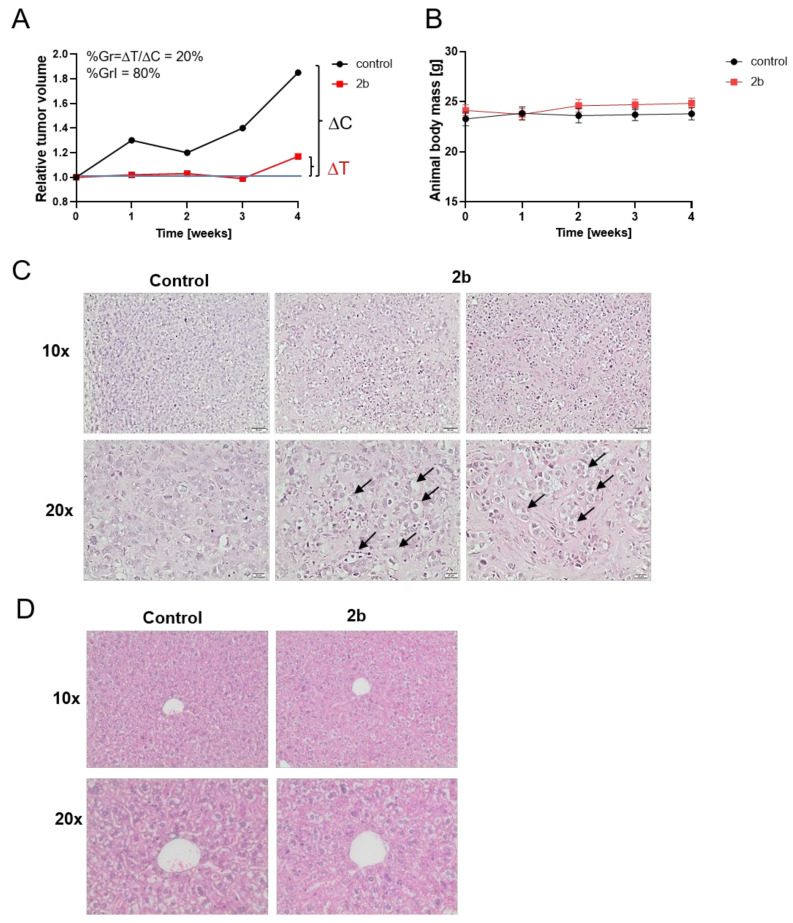
**2b** inhibits the growth of MCF-7 cell xenografts. Effect of **2b** (400 mg/mL) or vehicle (corn oil) treatment on the tumor volumes (**A**) and body weights of animals (**B**). Mean tumor volumes (n = 5) at a given treatment time point were calculated and expressed related to tumor volume at the beginning of treatment (taken as 1). Percent tumor growth (%Gr) and percent growth inhibition (%GrI) were calculated as indicated in Material and Methods. Histology of tumor (**C**) or liver (**D**) sections in control and **2b**-treated mice. Images were taken under 10× or 20× objective and representative results are shown. Tissue sections were fixed, embedded in paraffin, sectioned, and processed for H&E. Arrows indicate examples of cytoplasmic vacuolization.

Results presented in this work and our previously published data [25] indicate that **2b** is a more potent antiproliferative agent against cancer cells, including MCF-7 breast cancer cells, than UA, and at the same time is quite safe for normal cells. To elucidate if **2b** retains these features in vivo we tested it in mice models. First, the acute toxicology tests based on the oral administration have been performed using laboratory strain BALB/c mice which allowed a determination of the Maximum Tolerated Doses (MTD). MTD for UA (used as a positive control) was 200 mg/kg and for **2b**—400 mg/kg, which indicates that **2b** is less toxic to animals than UA (data not shown). Next, the effect of **2b** on the growth of MCF-7 xenograft in nude mice has been tested. As shown in Figure 8A, orally administered **2b** inhibited tumor growth and this was a cytostatic effect (ΔT ≥ 0). The percent tumor growth unaffected by treatment was calculated as 20% (%Gr) and inhibited by **2b**—as 80% (%GrI). The tested derivative did not affect body mass (Figure 8B). Similarly to in vitro cell culture, **2b** induced vacuolization of MCF-7 cells in tumor cells (Figure 8C). Importantly, vacuolization was not observed in livers either of control or **2b**-treated animals (Figure 8D).

## 3. Discussion

Usnic acid is widely explored in anticancer research; however, due to its moderate antiproliferative activity and toxicity to normal cells, the efforts of researchers have concentrated on modification of UA’s structure to improve its biological properties. In our previous study [25], we identified UA derivative **2b**, which inhibited the viability of breast, prostate and cervix cancer cells in concentrations 10 times lower than the parent compound, UA; at the same time, noncancerous cells were quite resistant. The present work shows that **2b** induced ER stress in MCF-7 breast cancer cells, which led to ER dilatation, massive cytoplasmic vacuolization and cell death, with features of paraptosis. The UPR response was evident at the transcriptome level and supported by immunoblotting for crucial ER stress regulators, namely BiP, IRE1α and GADD153 (CHOP). Moreover, cycloheximide, a translation inhibitor, protected against **2b**-induced vacuolization.

It has been reported that UA-induced toxicity in hepatic cells is mediated by ER stress and disturbance of calcium homeostasis [27]. The authors showed that the elevation of cytosolic Ca^2+^ by UA (6.25–50 μM) was caused by activation of store-operated Ca^2+^ entry (SOCE) channels and enhanced Ca^2+^ influx to the cells. UA upregulated STIM1 and ORAI1, key components of the SOCE system, and knockdown of ORAI1 prevented ER stress as well as ATP depletion in UA-treated HepG2 cells. Our results indicated that the increase in cytosolic Ca^2+^ ions was due to their release from ER stores through IP3R activation rather than calcium influx through SOCE. Multiple lines of evidence support this notion. Firstly, the elevation of cytosolic Ca^2+^ and the drop in cell viability were partially reversed by 2-APB. This is a cell-permeable, allosteric inhibitor of IP3-induced Ca^2+^ release. It has been shown that at higher concentrations, it may block store-operated Ca^2+^ entry (SOCE) channels as well [30]. However, in our model, BAPTA, which is a selective chelator of extracellular calcium, had no effect on **2b**-induced changes in cytosolic calcium levels or cell viability; therefore, we concluded that the elevated cytosolic Ca^2+^ level is of ER origin. Moreover, transcriptomic analysis indicated that **2b** downregulated the ORAI1 and STIM1 genes (logFC= −0.57 and −0.33 after 6 h; −1.9 and −2.3 after 24 h, respectively). Secondly, inhibition of the PKC responsible for IP3 generation protected against an increase in the levels of ER stress markers and partly blocked a drop in cell viability. Thirdly, the release of Ca^2+^ by the ER is a well-known inducer of ER stress and UPR [31] and occurs either by IP3R or the ryanodine receptor (RyR) [32]. In our model, **2b** mainly activated IP3Rs, which are more common than RyRs (RyRs are present predominantly in muscle, pancreatic and liver cells), and dantrolene, an RyRs inhibitor, had no effect on vacuolization and cell viability in our model.

IP3R-mediated release of Ca^2+^ from intracellular stores plays a key role in diverse processes, including cell proliferation, apoptosis, secretion, metabolism, migration and contraction, and is involved in multiple diseases such as cancer, pancreatitis or neurodegenerative diseases [8,33]. IP3 is generated from PIP2 by PLC [29,34]. The mammalian PLC family comprises a related group of complex, multidomain enzymes which cover a broad spectrum of regulatory interactions, including direct binding to G protein subunits, small GTPases from the Rho and Ras families, receptor and nonreceptor tyrosine kinases, and lipid components of cellular membranes [29,34]. Derivative **2b** may act on any of these molecules, which leads to PLC activation and enhanced generation of IP3, and thus activation of the IP3 receptors on the ER. Another possibility is that **2b** acts on other regulators of IP3Rs. Modulation of IP3R activity occurs by three main mechanisms: the local environment (pH, ATP and Mg^2+^ concentration), its phosphorylation status and regulatory proteins that directly affect these receptors, such as Bcl-2 family members, BiP or ERp44 [35]. The anti-apoptotic Bcl-2 protein targets the modulatory domain of IP3R, thereby suppressing excessive IP3R activity and protecting against Ca^2+^ release [36]. Our previous report showed that Bcl-2 protein levels were reduced in MCF-7 cells treated with **2b** [25], and the results of this work indicated that **2b** also reduces Bcl-2 mRNA (log FC= −4.1, after 24 h of treatment) (Supplementary data Table S2). The exact mechanism underlying the IP3R-mediated release of Ca^2+^ is currently under investigation.

Paraptosis is a type of nonapoptotic cell death featuring the formation of cytoplasmic vacuoles [37]. Swelling and vacuolization of the ER and/or mitochondria is mediated by mitogen-activated kinases and inhibited by AIP-1/Alix or cycloheximide [38,39]. This happens in the absence of caspase activation and other apoptotic markers, such as significant cell membrane blebbing or nuclear shrinkage and pyknosis [40,41]. Paraptosis is often accompanied by an alteration of Ca^2+^ and disruption of redox or ER homeostasis [42]. Not all these features are always observed in cells undergoing paraptosis, and thus the term “paraptosis-like cell death” is used when cell death resembles paraptosis but lacks some of its symptoms [43,44]. Many natural products, as well as developed compounds, have also been shown to induce paraptosis in cancer cells by disruption of calcium homeostasis [42]. Maintenance of calcium homeostasis in the ER is crucial for protein folding and the functioning of enzymes and chaperones [45], and its release by IP3Rs or RyRs may lead to the accumulation of misfolded proteins within the ER lumen that exert an osmotic force for the influx of water from the cytoplasm, and thus ER swelling [46]. Long-term ER stress may trigger fusion among swollen ER membranes and irreversible vacuolation, resulting in cell death [47], usually by paraptosis or apoptosis. In our model, we observed features of both cell death modes: the morphology of paraptotic death (vacuoles of the ER but not the mitochondrial origin, ER stress, no blebbing or chromatin condensation) and apoptosis induction (caspase-mediated PARP cleavage, a drop in Bcl-2, PS externalization) (this work and [25]). Comparable results have been observed previously in breast cancer cells; examples include treatment with the synthetic triterpenoid CDDO-Me, withaferin A or cancer cells of a different origin treated by celastrol [48,49,50].

Whatever kind of cell death is induced by **2b**, the important result of this work is that **2b** effectively inhibited breast tumour growth in vivo. Administered orally three times a week, **2b** revealed anticancer activity and—similar to the results in in vitro cultures—induced cancer cell vacuolization, which might become a marker of its activity. Importantly, treatment of animals with **2b** neither had adverse effects nor affected the morphology of healthy organs. These features make derivative **2b** a promising candidate for future research on its use for treatment of breast cancer patients.

## 4. Materials and Methods

### 4.1. Reagents

The procedures for the synthesis of usnic acid isoxazole **2b** have been described in [25]. RPMI 1640, DMEM, MEM, foetal bovine serum, the penicillin–streptomycin antibiotic mixture and trypsin-EDTA solution were from Corning, NY, USA. DMSO, hydrocortisone and thiazolyl blue tetrazolium bromide (MTT) were purchased from Sigma Aldrich (St. Louis, MO, USA). Insulin was from Thermo Fisher Scientific (Waltham, MA, USA). Antibodies against GRP78/BiP, and anti-rabbit, anti-mouse, anti-β-actin antibodies conjugated with horseradish peroxidase (HPR) were from Sigma Aldrich. Antibodies against ΙΡΕ1α and GADD153/CHOP were from Santa Cruz Biotechnology (Santa Cruz, CA, USA); an antibody against PARP was from Cell Signaling Technology (Danvers, MA, USA). The inhibitors 2-aminoethoxydiphenylborane (2-APB); 1,2-bis(2-aminophenoxy)ethane-*N*,*N*,*N*′,*N*′-tetraacetic acid (BAPTA); 1-[[[5-(4-nitrophenyl)-2-furanyl]methylene]amino]-2,4-imidazolinedione (dantrolene) and 1-[6-[[(17β)-3-methoxyestra-1,3,5(10)-trien-17-yl]amino]hexyl]-1*H*-pyrrole-2,5-dione (U-73122), and the mounting medium Fluoromount G and 4′,6-diamidino-2-phenylindole (DAPI) were from Sigma Aldrich. Cycloheximide was from Cayman Chemical (Ann Arbor, MI, USA); sodium phenylbutyrate (4-PBA) was from Santa Cruz Biotechnology. Baculovirus stock encoding RFP fused with the ER-targeting and retention sequences CellLight^®^ ER-RFP BacMam 2.0, MitoTracker Orange CMTMRos and Hoechst 33,342 were obtained from Thermo Fisher Scientific.

### 4.2. Cell Culture Conditions

The human breast adenocarcinoma cell line MCF-7 was from CLS Cell Lines Service GmbH (Eppelheim, Germany), human breast adenocarcinoma MDA MB 231 cells were obtained from Hirszfeld Institute of Immunology and Experimental Therapy Polish Academy of Sciences (Poland) and HB2 cells were provided by Dr. R. Sądej from Intercollegiate Faculty of Biotechnology, University of Gdansk and Medical University of Gdansk, Poland.

Monolayer cultures of MCF-7 and HB2 cell lines were maintained in RPMI 1640 or DMEM medium, respectively, supplemented with 10% (*v*/*v*) foetal bovine serum, a penicillin–streptomycin mixture and, in the case of HB2 cells, with 5 μg/mL hydrocortisone and 10 μg/mL bovine insulin. MDA-MB-231 cells were maintained in MEM medium supplemented with 10% foetal bovine serum, 0.1 mM nonessential amino acids and 1 mM sodium pyruvate. Each cell line was maintained at 37 °C in a humidified atmosphere with 5% CO_2_.

### 4.3. Cell Viability Assay

Cell viability was determined by the MTT method. Cells were seeded at a density of 4 × 10^3^ per well of a 96-well plate and allowed to attach overnight. The medium was replaced with fresh medium supplemented with the desired concentrations of **2b** for 24 h. In some experiments, cells were pretreated for 1 h with inhibitors: 1 or 10 μg/mL cycloheximide, 30 μM 2-ABP, 50 μM dantrolene, 10 μM BAPTA, 5 μM U-73122, and 3 or 4 mM 4-PBA.

Before the end of the treatment, 25 µL of the MTT solution (4 mg/mL) was added to each well. After 3 h of incubation, the medium was removed, and formazan crystals were dissolved in 100 µL of DMSO. Absorbance was measured at 570 nm (with a reference wavelength of 660 nm) in a Victor^3^ microplate reader. Data were obtained from at least 3 independent experiments performed in triplicate.

### 4.4. Immunofluorescence and Light Microscopy

For visualization of the ER, cells (2.5 × 10^4^) were plated on coverslips in 12-well plates and allowed to attach overnight. The next day, the cells were transfected with baculovirus encoding the ER signal sequence of calreticulin and KDEL (an ER retention signal) fused with TagRFP for specific ER fluorescence (CellLight^®^ ER-RFP BacMam 2.0, Thermo Fisher Scientific, USA). The transfection was performed according to the manufacturer’s instructions at PPC (particles per cell) = 50. After 48 h, the transfection was repeated; 48 h after the second transfections, cells were treated with either DMSO (control) or 3 μg/mL of **2b** for 18 h at 37 °C. At the end of the treatment, cells were stained with Hoechst 33,342 (5 μg/mL) for 15 min. After washing with warm PBS, cells were imaged under a fluorescence microscope (DMI4000B, Leica, Germany) under white light with difference interference contrast (DIC) and under fluorescence with appropriate filter sets. Cells not transfected with baculovirus and treated with a vehicle (DMSO) or **2b** served as a reference to exclude the impact of transfection on the cells’ response to **2b**.

For visualization of the mitochondria, cells were plated on coverslips in 12-well plates and allowed to attach overnight. The next day, the cells were treated with a vehicle (DMSO; control) or **2b** (3 μg/mL) for the indicated time. At the end of the treatment, a mitochondria-specific dye, MitoTracker Orange CMTMRos, was added to the growth medium to a concentration of 100 nM for 1 h. Next, cells were washed with warm PBS and fixed with 2% paraformaldehyde. After rinsing several times with the buffer, cells were stained with DAPI, followed by washing. The coverslips were mounted with Fluoromount G mounting medium (Sigma Aldrich, USA). Cells were analysed under a fluorescence microscope in fluorescence with appropriate filter sets.

### 4.5. Transmission Electron Microscopy (TEM)

Transmission electron microscopy of MCF-7 cells was performed as described previously [25]. Briefly, cells (2 × 10^5^) were plated in 12-well plates and allowed to attach overnight. Next, cells were treated with either DMSO (control), or 1 or 3 μg/mL of **2b** for 24 h at 37 °C. For TEM, cells were fixed in ice-cold 2.5% electron microscopy-grade glutaraldehyde (Polysciences) in 0.1 M PBS (pH 7.4). The samples were rinsed with PBS, post-fixed in 1% osmium tetroxide with 0.1% potassium ferricyanide, dehydrated through a graded series of ethanol washes (30–100%), and embedded in Epon (Fluka, Buchs, Switzerland). Semi-thin (300 nm) sections were cut using an RMC Power Tome XL ultramicrotome, stained with 0.5% toluidine blue and examined under a light microscope. Ultrathin sections (45 nm) were stained with 2% uranyl acetate and Reynold’s lead citrate, and examined on a Philips CM100 transmission electron microscope.

### 4.6. RNA Library Preparation and High-Throughput Sequencing

For RNA isolation, cells were seeded in 10 cm plates (1 × 10^6^) and treated with 3 μg/mL **2b** or DMSO for 6 or 24 h. Total RNA was isolated using a High Pure RNA Isolation Kit (Roche Diagnostics, Warszawa, Poland) according to the manufacturer’s instructions. The concentration and quality of isolated RNA were determined using an Agilent 2100 Bioanalyzer (Agilent Technologies, Santa Clara, CA, USA). RIN values were > 8.0. The library construction and RNA sequencing were performed by the RNA-Seq Service (Macrogen, Seoul, Korea).

### 4.7. Read Mapping and Gene Expression Profiling

Sequencing quality was evaluated using FastQC (v0.11.7, https://github.com/s-andrews/FastQC (accessed on 25 February 2020)) and, due to the overall high quality of the generated reads, was deemed unnecessary. Reads were next mapped to the reference human genome obtained from GENCODE (release 30, GRCh38 primary assembly) using the STAR RNA-seq aligner [51] version 2.6.0a. First, a genome index was generated, incorporating the gene annotation file for the process (GENCODE release 30, comprehensive gene annotation GTF file). All reads were then aligned to the genome in two-pass mode, with the rest of the parameters set to the default, except for:—*readFilesCommand* zcat, —*outSAMtype* BAM SortedByCoordinate, —*quantMode* GeneCounts. The obtained counts in the “ReadsPerGene.out.tab” files, which coincided with the results of the htseq-count tool [52], were used to generate a raw count matrix of uniquely mapped per-gene counts.

Differential gene expression analysis was conducted using the edgeR package version 3.26.8 [53]. Low-expression genes were filtered out (i.e., we required >1 count per million in at least 2 samples) and normalized using the trimmed mean of M-values (TMM) normalization method [54]. Statistical tests to identify differentially expressed genes were then conducted by applying the generalized linear model (GLM, *glmQLFit* function in edgeR) for comparing the treated and untreated samples at different time points and concentrations, and using the available biological replicates. Benjamini–Hochberg correction of raw *p*-values was applied to the final differential expression results to correct for multiple hypothesis testing, and we picked the significant genes as those fulfilling the threshold of FDR  < 0.05 and a log2 fold change ≥ 1.

Data processing, as well as visualization of the generated results, was performed in the R environment (version 3.6.3, https://www.r-project.org/ (accessed on 25 February 2020)). For drawing expression heatmaps, the “pheatmap” package (version 1.0.12) was used.

### 4.8. Gene Ontology and KEGG Pathway Enrichment Analyses

We analysed the identified significant differentially expressed genes from all compared setups for the enrichment of Gene Ontology terms (biological process, BP; molecular function, MF; cellular component, CC) (http://geneontology.org/ (accessed on 25 February 2020)) and KEGG pathways (Kyoto Encyclopedia of Genes and Genomes, https://www.genome.jp/kegg/pathway.html (accessed on 25 February 2020)). The analyses were performed within the “limma” package (version 3.40.6) with the *goana* and *kegga* functions. The results were further cross-checked with those obtained from the online platform Enrichr (https://maayanlab.cloud/Enrichr/, (accessed on 30 March 2020)) [55]). Pathway expression visualization was performed using Pathview Web [56,57]. The results generated within the course of this study can be found in the NCBI Gene Expression Omnibus under accession number GSE191314 (https://www.ncbi.nlm.nih.gov/geo/query/acc.cgi?acc=GSE191314 (accessed on 20 December 2021)).

### 4.9. Immunoblotting

Cells were treated with **2b** and lysed using a solution containing 50 mM Tris (pH 7.5), 1% Triton X-100, 150 mM NaCl, 0.5 mM EDTA, protease and phosphatase inhibitor cocktails (Roche Diagnostics). The lysates were cleared by centrifugation. Proteins were separated by SDS-PAGE and transferred onto a PVDF membrane. The membrane was blocked with 5% nonfat dry milk in phosphate-buffered saline and incubated with the desired primary antibody overnight at 4 °C. The membrane was then treated with the appropriate secondary antibody for 1 h at room temperature. Immunoreactive bands were detected with an enhanced chemiluminescence reagent (Thermo Fisher Scientific). Blots were stripped and reprobed with anti-actin antibodies to normalize them for differences in protein loading. Each protein was detected 2 or 3 times in independently prepared lysates. Densitometry analysis was carried out using Quantity One 1-D Analysis software (Bio-Rad, Hercules, CA, USA).

### 4.10. Measurement of Ca^2+^ Level

Cells were seeded at a density of 2 × 10^4^ per well of a 96-well plate and allowed to attach overnight. The medium was replaced with fresh medium supplemented with the desired concentrations of **2b** for 6 h, and Ca^2+^ levels were evaluated using the Fluo-4 Direct Assay Kit (Invitrogen) according to the producer’s instructions.

### 4.11. Animal Studies

The experiments on mice were conducted at the Tri-City Academic Laboratory Animal Centre. The animal protocol was approved by the Local Ethics Committee for Animal Experimentation in Bydgoszcz (permit No. 20/2019). Animal experimentation was performed in accordance with EU directive 2010/63/EU. Female nude mice (ATHYM-foxn1nu/nu, 4 weeks old) were purchased from Janvier Labs (Le Genest-Saint-Isle, France). At 6 weeks of age, to generate tumour xenografts, 1 × 10^6^ of MCF-7 cells in Matrigel were injected subcutaneously into the flanks of each mouse. When the tumour volume reached up to approximately 80 mm^3^, mice were randomly divided into 3 groups (n = 5 in each group). Animals were treated 3 times a week for 4 weeks by oral gavage with corn oil (control group) or **2b** suspended in corn oil (400 mg/kg). Tumour growth and body weight were recorded every 2 days. At the end of the experiment, mice were sacrificed, then tumours and livers were excised, measured and stored for further analysis. The treatment efficacy was calculated as the percentage of tumour growth (%Gr = 100 × ΔT/ΔC, where ΔT = T − T_0_ and ΔC= C − C_0_) and the percentage of growth inhibition (%GrI = 100 − %Gr) according to [58].

### 4.12. Histopathology

Dissected tumours and livers were fixed with 4% paraformaldehyde in PBS and paraffin-embedded. The 5-µm-thick sections were mounted on Superfrost Plus adhesive slides (Thermo Fisher Scientific, Waltham, MA, USA). All samples were stained with haematoxylin and eosin (H&E, Eosin Y, Harris Hematoxylin Shandon, Thermo Scientific, USA) to determine tissue structure and the degree of vacuolization. Slides were mounted with DPX (Fluka, Buchs, Switzerland). All comparative sections were performed at the same time under identical conditions. Images were taken using an Olympus light microscope IX51 with a CCD camera and CellSens software.

### 4.13. Statistical Analysis

All data are shown as the means ± standard error (SE) of at least 3 independent experiments. The significance of differences between the control and treated cells or between tested variants was analysed with ANOVA and Dunnett’s or Tukey’s multiple comparisons post-hoc tests, respectively, using GraphPad Prism (version 8). Differences were considered significant at *p* < 0.05 and are marked with an asterisk.

## 5. Conclusions

This work shows that the derivative of usnic acid (a secondary lichen metabolite) named **2b** induced ER stress in breast cancer cells and not in healthy cells. It was caused by the elevated release of Ca^2+^ from ER and led to cancer cells’ death, with features of apoptosis and paraptosis. Derivative **2b** also effectively blocked tumour growth in vivo. In animals treated with **2b**, cancer cells revealed cytoplasmic vacuolization which was not observed in their liver cells.

## Figures and Tables

**Table 1:** The most significantly enriched pathways identified by KEGG analysis in MCF-7 cells treated with **2b** (3 μg/mL) for 6 h in comparison with untreated cells.

No.	Pathway	N	Up	Down	P.Up	P.Down
hsa04141	Protein processing in endoplasmic reticulum	151	87	20	8.22 × 10^−19^	1
hsa04015	Rap1 signalling pathway	139	23	61	0.99	4.86 × 10^−9^
hsa04390	Hippo signalling pathway	123	15	53	1	1.02 × 10^−7^
hsa04360	Axon guidance	146	14	60	1	1.19 × 10^−7^
hsa03060	Protein export	23	16	0	5.09 × 10^−6^	1
hsa04550	Signalling pathways regulating the pluripotency of stem cells	104	19	43	0.94	5.88 × 10^−6^
hsa03008	Ribosome biogenesis in eukaryotes	70	33	8	2.09 × 10^−5^	0.99
hsa00510	N-glycan biosynthesis	45	24	8	2.17 × 10^−5^	0.80
hsa04750	Inflammatory mediator regulation of TRP channels	67	9	30	0.99	2.39 × 10^−5^
hsa00970	Aminoacyl-tRNA biosynthesis	44	22	3	1.68 × 10^−4^	1
hsa05200	Pathways in cancer	368	78	109	0.92	2.60 × 10^−4^
hsa00513	Various types of N-glycan biosynthesis	35	18	3	4.19 × 10^−4^	0.99
hsa04916	Melanogenesis	71	12	28	0.95	6.02 × 10^−4^
hsa05418	Fluid shear stress and atherosclerosis	100	24	36	0.55	8.44 × 10^−4^
hsa04914	Progesterone-mediated oocyte maturation	76	13	29	0.95	9.11 × 10^−4^

**Table 2:** The most significantly enriched pathways identified by KEGG analysis in MCF−7 cells treated with **2b** (3 μg/mL) for 24 h in comparison with untreated cells.

No.	Pathway	N	Up	Down	P.Up	P.Down
hsa04141	Protein processing in endoplasmic reticulum	151	109	30	6.97 × 10^−15^	1
hsa04015	Rap1 signalling pathway	139	22	98	1	1.64 × 10^−10^
hsa04810	Regulation of actin cytoskeleton	152	31	101	1	1.44 × 10^−8^
hsa04360	Axon guidance	146	27	97	1	2.80 × 10^−8^
hsa04390	Hippo signalling pathway	123	23	84	1	3.54 × 10^−8^
hsa05168	Herpes simplex virus 1 infection	372	204	111	3.96 × 10^−8^	1
hsa05200	Pathways in cancer	368	108	208	1	5.26 × 10^-7^
hsa04750	Inflammatory mediator regulation of TRP channels	67	13	47	1	1.20 × 10^−5^
hsa05205	Proteoglycans in cancer	156	37	95	1	1.28 × 10^−5^
hsa05206	MicroRNAs in cancer	141	43	86	1	2.98 × 10^−5^
hsa01100	Metabolic pathways	1091	385	541	1	4.27 × 10^−5^
hsa03040	Spliceosome	129	75	32	6.43 × 10^−5^	1
hsa05322	Systemic lupus erythematosus	47	9	34	1	7.18 × 10^−5^
hsa04611	Platelet activation	83	18	54	1	7.85 × 10^−5^
hsa05100	Bacterial invasion of epithelial cells	65	17	44	1	8.89 × 10^−5^
hsa05225	Hepatocellular carcinoma	138	41	82	1	1.62 × 10^−4^
hsa05412	Arrhythmogenic right ventricular cardiomyopathy (ARVC)	43	9	31	1	1.66 × 10^−4^
hsa00970	Aminoacyl-tRNA biosynthesis	44	30	9	2.52 × 10^−4^	1
hsa04916	Melanogenesis	71	15	46	1	3.02 × 10^−4^
hsa04713	Circadian entrainment	60	10	40	1	3.04 × 10^−4^

## Data Availability

Data and access to the public database are contained within the article.

## Supplementary Materials

The following are available online at https://www.mdpi.com/article/10.3390/ijms23031802/s1.
